# Genome and Transcriptome Sequences Reveal the Specific Parasitism of the Nematophagous *Purpureocillium lilacinum* 36-1

**DOI:** 10.3389/fmicb.2016.01084

**Published:** 2016-07-19

**Authors:** Jialian Xie, Shaojun Li, Chenmi Mo, Xueqiong Xiao, Deliang Peng, Gaofeng Wang, Yannong Xiao

**Affiliations:** ^1^Key Laboratory of Plant Pathology of Hubei Province, College of Plant Science and Technology, Huazhong Agricultural UniversityWuhan, China; ^2^State Key Laboratory for Biology of Plant Diseases and Insect Pests, Institute of Plant Protection, Chinese Academy of Agricultural SciencesBeijing, China

**Keywords:** *Purpureocillium lilacinum*, parasitism, nematophagous fungi, appressorium, genome, transcriptome

## Abstract

*Purpureocillium lilacinum* is a promising nematophagous ascomycete able to adapt diverse environments and it is also an opportunistic fungus that infects humans. A microbial inoculant of *P. lilacinum* has been registered to control plant parasitic nematodes. However, the molecular mechanism of the toxicological processes is still unclear because of the relatively few reports on the subject. In this study, using Illumina paired-end sequencing, the draft genome sequence and the transcriptome of *P. lilacinum* strain 36-1 infecting nematode-eggs were determined. Whole genome alignment indicated that *P. lilacinum* 36-1 possessed a more dynamic genome in comparison with *P. lilacinum* India strain. Moreover, a phylogenetic analysis showed that the *P. lilacinum* 36-1 had a closer relation to entomophagous fungi. The protein-coding genes in *P. lilacinum* 36-1 occurred much more frequently than they did in other fungi, which was a result of the depletion of repeat-induced point mutations (RIP). Comparative genome and transcriptome analyses revealed the genes that were involved in pathogenicity, particularly in the recognition, adhesion of nematode-eggs, downstream signal transduction pathways and hydrolase genes. By contrast, certain numbers of cellulose and xylan degradation genes and a lack of polysaccharide lyase genes showed the potential of *P. lilacinum* 36-1 as an endophyte. Notably, the expression of appressorium-formation and antioxidants-related genes exhibited similar infection patterns in *P. lilacinum* strain 36-1 to those of the model entomophagous fungi *Metarhizium* spp. These results uncovered the specific parasitism of *P. lilacinum* and presented the genes responsible for the infection of nematode-eggs.

## Introduction

Parasitic nematodes cause great loss to agriculture, leading to a loss of US$157 billion each year around the world (Abad et al., 2008). For decades, nematode control has largely depended on various chemical nematicides. However, chemical control usually leads to environmental side effects and human health risks (Li et al., 2015). Some bio-control agents such as soil *hyphomycetes* show great promise. The egg-pathogenic fungus *Purpureocillium lilacinum* (*P. lilacinum*), which was previously known as *Paecilomyces lilacinus*, is one of the most widely reported entophytic filamentous fungi used as a bio-control agent to control root-knot nematodes. One of its strains (PL251) has being registered as a biological nematicide in the USA (Kiewnick et al., 2011). *P. lilacinum* is frequently isolated from soil, forests, grass, nematodes, and insects (Luangsa-ard et al., 2011). In addition, the fungus has been detected in the rhizosphere of many crops (Lopez et al., 2014). It is also an opportunistic pathogen of immunodeficient humans and other vertebrates (Inglis et al., 2005).

*P. lilacinum* can grow in a wide range of temperatures with the optimal growth range from 26 to 30°C (Banu et al., 2006). This organism is tolerant to broad range of pH values and can grow on a variety of carbon and nitrogen media (Banu et al., 2006; Sun and Liu, 2006). Moreover, *P. lilacinum* is well-known for producing all types of proteolytic enzymes and carbohydrate hydrolases (such as serine proteases and chitinases), which can destroy the lipid and chitin layers of nematode eggshell (Wang et al., 2010). Besides, *P. lilacinum* can also produce various biologically active secondary metabolites including polyketides and non-ribosom-synthesized peptides, such as leucinostatins, which exert a range of biological activities including nematicidal, anti-viral, anti-tumor, and phytotoxicity properties (Mori et al., 1982; Park et al., 2004; Ishiyama et al., 2009).

Gene functional studies of *P. lilacinum* have identified a few genes that are involved in virulence and fungal development (Wang et al., 2010; Yang et al., 2011). Most studies have focused on practical bio-control effects on crops and medical discoveries in human (Ramam, 2014; López-Medrano et al., 2015). The underlying molecular biological mechanism is still unknown. The genome of a *P. lilacinum* strain that was isolated from the tannery sludge in India was already sequenced. The report has summarized the fundamental characteristics of the fungus, but the genes related to pathogenesis were not well-explored (Prasad et al., 2015). Here, we sequenced another *P. lilacinum* strain 36-1, which was specifically isolated from nematode eggs, and performed a comparative analysis with the other sequenced genome of *Ascomycetes*, especially for nematophagous or entomopathogenic fungi. The comparison revealed a series of gene family expansions and contractions in *P. lilacinum* 36-1 that could distinguish it from plant pathogens, as well as species-specific genes gain or loss that implemented different pathogenic strategies. Moreover, the transcriptional responses of *P. lilacinum* strain 36-1 to *Meloidogyne incongnita* eggs were explored by RNA-seq technique and the genes involved in pathogenicity were demonstrated.

## Materials and methods

### Fungus strains

*P. lilacinum* strain 36-1 was isolated from the surface of the eggs of *Meloidogyne incognita* in the field soil from Hubei Province in China (Yang et al., 2015). The fungus were cultured in potato dextrose agar (PDA) medium at 28°C. The conidia of *P. lilacinum* strain 36-1 were suspended in MM liquid medium, which contained 1g L^−1^ NH_4_NO_3_, 0.5g L^−1^ KH_2_PO_3_, 1.5g L^−1^ K_2_HPO_3_, 1.0 g L^−1^ NaCl, and MgSO_4_.7H_2_O.

### Genome sequencing and assembly

The genome of *P. lilacinum* strain 36-1 was shotgun-sequenced with the Illumina next generation sequencing technology. DNA libraries with 500 and 6000 bp insertions were constructed and sequenced with Hiseq2000 at BGI (Shenzhen, China). The genome sequence was assembled using SOAPdenovo 1.05 (Li et al., 2010).

### Gene prediction and annotations

The gene modes were predicted with a combined method in priority order. Gene structures were generated by Genewise 2.20 (Birney et al., 2004) using protein sequences from *Metarhizium anisopliae, Metarhizium acridum, Ophiocordyceps sinensis, Trichoderma virens*, and *Aspergillus nidulans* as references, with the ab initio predictor Augustus 2.6.1 (Keller et al., 2011) and SNAP (Korf, 2004) using annotated information from *A. nidulans* FGSC A4, GeneMark-ES 2.3e (Borodovsky and Lomsadze, 2011). The final gene annotation set was incorporated and redundancies were removed. Transfer RNAs (tRNAs) were predicted with tRNAscan-SE (Schattner et al., 2005). Secreted proteins were identified with SignalP3.0 using a hidden Markov model (Bendtsen et al., 2004). Transmembrane proteins were predicted with TMHMM 2.0 (Krogh et al., 2001). The subcellular localization of proteins were identified by Targetp1.1 (Emanuelsson et al., 2000). Transposon elements were analyzed with a combined method. RepeatMasker was used to Blast against the Repbase library, and a transposon model was constructed by building an XDFDatabase with *P. lilacinum* strain 36-1 genome sequences (Saha et al., 2008). Tandem repeat elements were identified by Tandem Repeat Finder (TRF) (Benson, 1999). The RIP (Repeat Induced Point mutation) index was determined with RIPCAL software by referencing them against the non-repetitive control families (Hane and Oliver, 2008). The whole genome alignment between *P. lilacinum* 36-1 and the India strain was performed using MAUVE aligner version 2.4.0 using progressive algorithm with default parameters prior to reordering the scaffolds by Mauve Contig Mover (MCM) (Darling et al., 2004). Locally collinear blocks (LCB) with lengths of at least 100 bp was displayed by R package genoPlotR (Guy et al., 2011). The genome segment rearrangements and SNPs were identified by reciprocal genomic comparison between *P. lilacinum* 36-1 and the India strain.

### Orthology and phylogenomic analysis

Orthologous proteins were identified with ProteinOrtho 5.08 following the default parameters (Lechner et al., 2011). Totally, 19 species from different ecological niches were selected, including the phytopathogenic fungi *Fusarium graminearum, Magnaporthe oryzae, Ustilaginoidea virens, Verticillium dahiliae, Rhizoctonia solani*, and *Ustilago maydis*; saprophytes *Neurospora crassa, T. reesei, T. virens*, and *Saccharomyces cerevisiae*; human pathogenic fungi *A. nidulans, A. fumigatus*, and *Candida albicans*; and nematophagous/entomophagous fungi *O. sinensis, Beauveria bassiana, M. acridum, M. anisopliae, Cordyceps militaris*, and *Pochonia chlamydosporia*. In sum, 580 single-copy orthologous proteins were acquired and aligned with muscle 3.8 (Edgar, 2004). The alignment sequences were concatenated and subsequently used for phylogenetic reconstruction by maximum likelihood method with the Dayhoff amino acid substitution model implemented in MEGACC7.0 (Molecular Evolutionary Genetics Analysis Computational Core) (http://www.megasoftware.net/) with 1000 bootstrap replications and complete-deletions for gaps or missing sites.

### Protein family classification and evolutionary analysis

Whole genome protein families were classified by InterProScan analysis (Jones et al., 2014). The peptidase families were identified by BLASTP searching against MEROPS peptidase database release 9.12 with a cutoff *e*-value of 1e-20 (Rawlings et al., 2014). The transporters were classified on the basis of Transport Classification Database (Saier et al., 2014). Carbohydrate-active enzymes were classified by using hmmer 3.0 (Mistry et al., 2013) to search against a library of catalytic and carbohydrate-binding module enzymes acquired from dbCAN (Yin et al., 2012). G-protein-coupled receptors were selected from the best hits with GPCRDB sequences (Horn et al., 2003) and by confirming that they contained seven transmembrane helices with the amino terminus outside and the carboxyl terminus inside the plasma membrane. Homologs of the Pth11-like GPCRs in *Magnaporthales* (Kulkarni et al., 2005) were identified by local BLASTP analysis with a cutoff *e*-value of 1e-10. The divergence time between species was estimated with the Langley-Fitch method with r8s (Marazzi et al., 2012) by calibrating against the reassessed origin of the Ascomycota at 500–650 million years ago (Lücking et al., 2009).

### Transcriptome mapping and expression analysis

*P. lilacinum* strain 36-1 was cultured on potato dextrose agar for 15 days. The conidia were washed out with 10 mL of sterile distilled water and adjusted to a concentration of 1 × 10^7^ cfu mL^−1^ with MM liquid medium. *M. incongnita* egg-masses were isolated from tomato roots and sterilized with 1% (v/v) NaClO. The dissociated eggs were suspended in sterile distilled water. The conidia that were precultured in MM liquid media for 24 h were mixed with water or *M. incongnita* eggs, the concentration of which was adjusted to 2 eggs per microliter. Each treatment was repeated three times. The mixtures were filtered to collect the mycelia after 24 h. The infection process was checked under a light microscope. All the repeat treatments were collected and mixed together following RNA extractions. Total RNAs were extracted according to standard protocol plus an incubation treatment with RNase-free DNase I. mRNAs were purified and then reverse-transcribed into cDNA for library construction. Each library product was sequenced using a library with a read insertion size of 200 bp by using Illumina GA II technology without technology replications. The paired-end reads were filtered as follows: Adaptor sequences were trimmed from the reads with the NGS QC toolkit (ver. 2.3) (Patel and Jain, 2012); and reads with < 80% “Q > 20” bases were removed. We used TopHat2 (ver. 2.1.1) (Trapnell et al., 2009) to map filtered mRNA reads onto the predicted genes model of the genome. The RNA expression count and identification of differentially expressed genes were performed using GFOLD v1.1.1 with a significant cutoff of 0.01 for the fold change. The software GFOLD was especially designed for RNA-Seq reads when no replicate was available, generalizing the reliable fold change by considering the posterior distribution of the log fold change (Feng et al., 2012). To reduce the false positive ratio, we used a stricter expression value (**|**log_2_ fold-change| > 2) to select differential expression genes. The gene ontology (GO) annotation of the genome was conducted with ipr2go by a local R script. The GO and KEGG enrichment were analyzed with GOstats in Bioconductor (Falcon and Gentleman, 2007).

### Growth profiles on a single carbon source

The MM medium and agar were mixed together as a basic culture medium in 9 cm Petri dishes. Xylan, cellulose or pectin were used as single carbon source to evaluate the growth abilities of different fungi with diverse carbon sources. Inoculations of *P. lilacinum* strain 36-1 and other fungi (*F. graminearum* and *T. reesei*) were placed on centrals sections of these mediums and cultured at 28°C for 3 days. Three biological replicates were set.

### qPCR analysis

The total RNAs used for RNA-Seq were also employed to analyze the expression levels of the target genes. First, the mRNAs were reverse-transcribed with SuperScript III Reverse Transcriptase (Invitrogen, Shanghai, China) by oligo dT according to the instructions. SsoFast™ EvaGreen Supermix (Bio-Rad, USA) was used to quantify the target genes by putting 5 ng of cDNA products into a 10 μL quantification system. The qPCR cycling parameters were 95°C for 20 s, then 45 cycles of 95°C for 10 s, and 60°C for 20 s. β-*Tubulin* was used as an endogenous reference gene (Yang et al., 2011). The experiments were conducted in quadruplicate. The 2^−ΔΔCt^ method was used to calculate the transcript abundance. The primers used for qPCR are listed in Table S1.

## Results

### General features

The genome of *P. lilacinum* strain 36-1 was shotgun sequenced up to 75 × coverage using Illumina paired-end sequencing with insertion sizes between 500 and 6000 bp. A draft genome sequence was assembled on the basis of the high-quality reads using the de novo assembler SOAPdenovo (Li et al., 2010). We ultimately obtained a 37.61 Mb genome that was assembled into 240 scaffolds with the N50 size being over 1.3 Mb (Table 1), which was a bit different from the *P. lilacinum* India strain because of the various sequencing methods and different sources of the fungus (Table 1). The GC contents of the assembled genome was 58.57%, which was the same as the previous sequenced *P. lilacinum* strain (Table 1). However, *P.lilacinum* 36-1 seemed to have a more compact genome as indicated by a series of features, such as higher gene density, longer average length of genes and more tRNAs (Table 1). The Whole genome alignment analyses showed large numbers of locally collinear blocks (LCB) between this two strains, but more genome segments rearrangements and SNPs indicated that the genome of *P. lilacinum* 36-1 was more dynamic than the India strain (Figure S1, Table 1).

**Table 1:** **Comparison of genome feature between two different strains of ***P. lilacinum*****.

**Features**	***P. lilacinum* strain 36-1**	***P. lilacinum* India strain**
District	Hubei province, China	Kanpur, India
Source	Nematode-eggs, soil	Tannery sludge rich in Cr
Genome size (Mb)	37.61	40.02
Coverage	75 ×	200 ×
Scaffold No.	240	301
Scaffold N50 (kb)	1328	1827
G+C content %	58.57	58.57
Repeat %	2.4	1.68
Protein-coding genes	13,150	13,266
Avg. Gene length (bp)	1933	1512
Gene density (No. gene per Mb)	346	303
tRNA	103	91
Insertions	14,176	2106
Deletions	1744	14,433
Inversions	3657	3526
SNPs (%)	1.4(521,424)	1.3(519,777)

*The genome rearrangement and SNPs were obtained by reciprocal comparison between this two P. lilacinum strains with MAUVE software*.

We further evaluated the quality of the genome assembly with BWA (Li and Durbin, 2009). As a result, 98.20% of the reads were mapped back to the assembly, which covered over 99.99% of the scaffolds, excluding the gaps. This finding showed that the current assembly covered almost all the genome sites. Moreover, ~97.46% of the assembly was covered by at least 20 × reads, representing the high level of accuracy for assembly at single nucleotide sites (Figure S2). Based on the extensive presence of conserved protein families in eukaryotes, the ratio of core eukaryotic genes (CEGs) was evaluated in the current assembly using CEGMA (Parra et al., 2007). Finally, 242 complete-partial CEGs were found in the current assembly, making up 97.58% the total CEGs. All of these results suggested a largely complete assembly.

**Figure 1:**
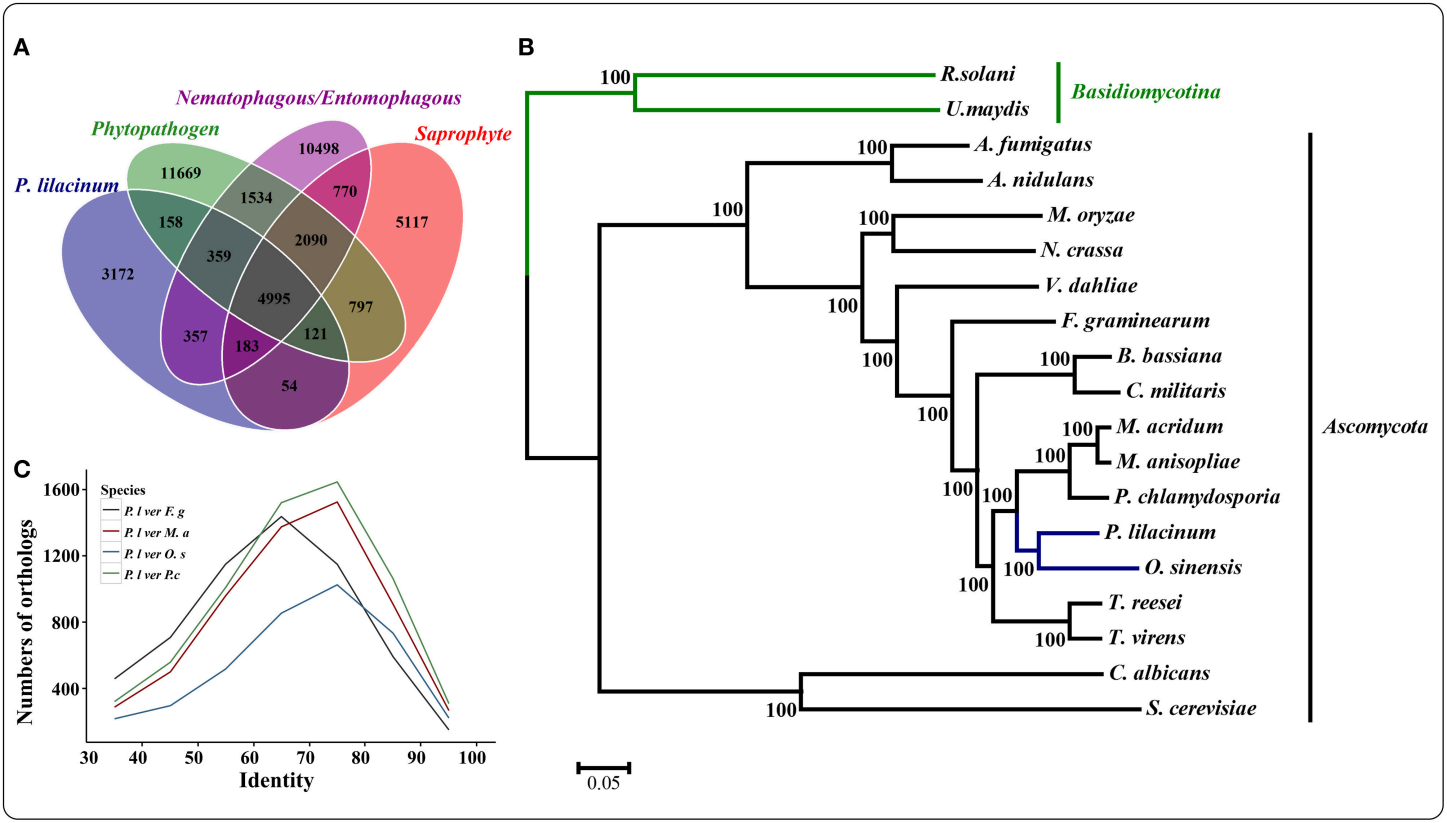
**Comparative genomics and evolutionary analysis of ***P. lilacinum*** strain ***36-1*****. **(A)** Numbers of homologous families between different fungi with different lifestyle. Phytopathogenic fungi include *Fusarium graminearum, Magnaporthe oryzae*, and *Ustilaginoidea virens*; Saprophytism include *Neurospora crassa, Trichoderma reesei*, and *Aspergillus nidulans*. Nematophagous/Entomophagous fungi include *Ophiocordyceps sinensis, Beauveria bassiana, Metarhizium acridum, Cordyceps militaris*, and *Pochonia chlamydosporia*; **(B)** A maximum likelihood phylogenomic tree constructed using the Dayhoff amino acid substitution model showing the evolutionary relationship of *P. lilacinum* strain 36-1 with other fungi; **(C)** Numbers of orthologous genes with different levels of protein identity between *P. lilacinum* strain 36-1 and other fungi. Identity refers to the reciprocal similarity (>30%) of proteins between *P. lilacinum* and other fungi by BLASTP.

The genome of *P. lilacinum* 36-1 was predicted to have 13150 protein coding genes, which was slightly lower than the number for India strain (Table 1) but higher than other sequenced fungi (Table S2). Relative to fungi in diverse ecological niches, *P. lilacinum* 36-1 had more line-specific genes with nematophagous/entomophagous fungi (357 genes) than phytopathogenic (158 genes) and saprophytic fungi (54 genes) (Figure 1A). Among the 357 genes, 187 could be annotated in the gene ontology (GO) database (*p* < 0.05), of which genes with functions of DNA binding, peptidases, kinase activity, oxidoreductase activity, transmembrane transporter activity, and ion binding activity were well-represented. Additionally, *P. lilacinum* 36-1 shared 4995 homologous genes with other sequenced fungi and had 3172 species-specific genes (Figure 1A). The orphans mapped onto GO database were primarily enriched in terms of metabolic regulation and cellular macromolecule biosynthetic processes (*p* < 0.05) (Table S3), suggesting a complex of physiological regulation activities in *P. lilacinum* 36-1.

A phylogenetic tree was constructed from 580 single-copy conserved orthologous groups acquired by comparing the protein sequences in 19 species. The results showed that *P. lilacinum* 36-1 was more closely related to the entomophagous fungus *O. sinensis* (Figure 1B). According to the Langley-Fitch method, we found that *P. lilacinum* 36-1 diverged before a split with *O. sinensis* 74~94 million years ago (MYA) (Figure 1B). However, these two fungi shared far few reciprocal best hit genes than those of the nematode eggs parasite fungus *P. chlamydosporia* and the entomophagous fungus *M. acridum* (Figure 1C).

**Figure 2:**
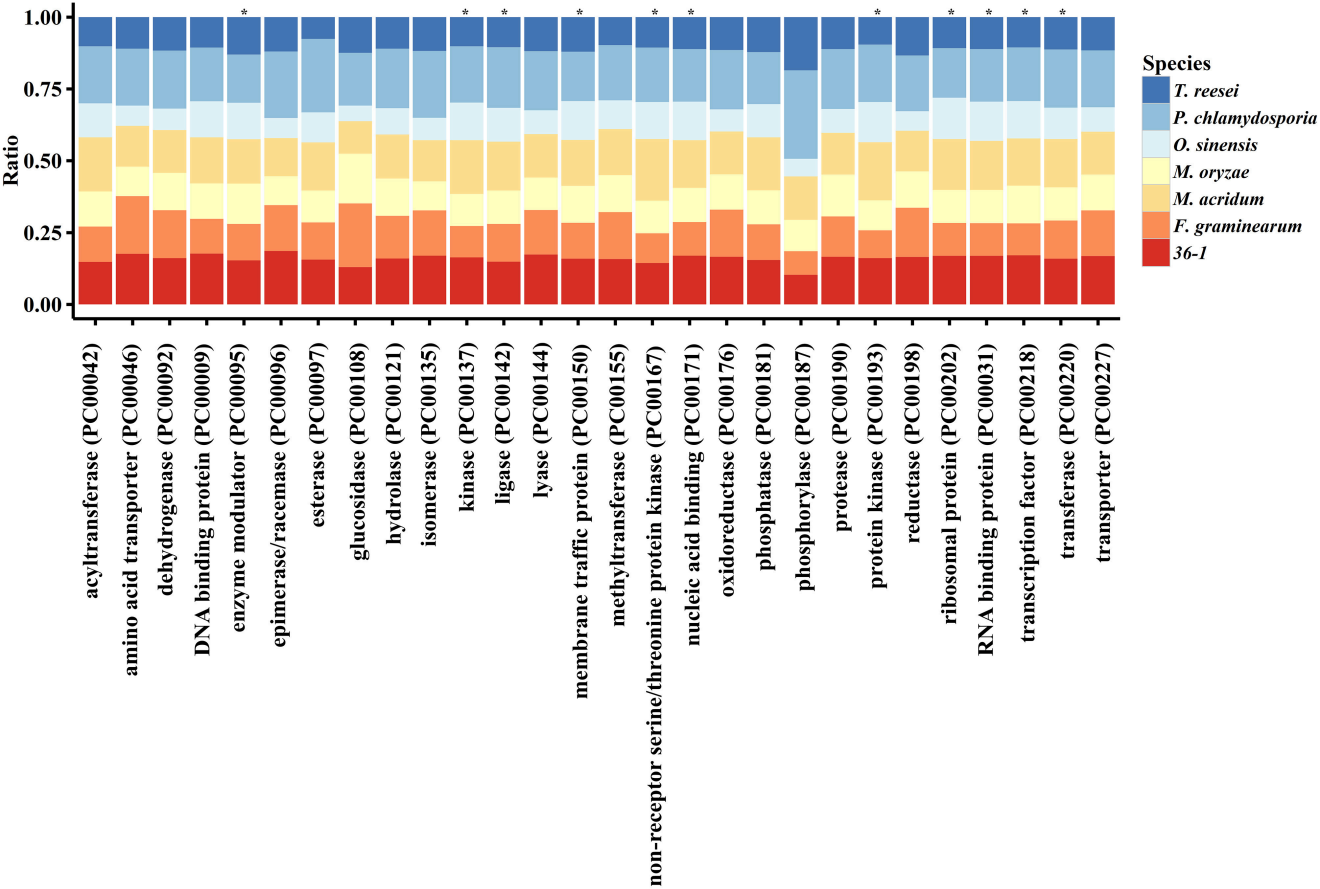
**Comparison of gene families between different fungi**. ^*^:Indicates significantly expansion of *P. lilacinum* 36-1 relative to *O. sinensis* (*P* < 0.05); Protein class indicates the classification system by PANTHER. Ratio indicates the percentage of protein class in different fungi.

PANTHER (Protein Analysis THrough Evolutionary Relationships, http://pantherdb.org) is widely used for comprehensive protein evolutionary and functional classification (Mi et al., 2016). By using a PANTHER analysis, we compared the profiles for gene expansions or contractions in seven fungi by statistical overrepresentation test with a Bonferroni correction. In comparison with *O. sinensis, P. lilacinum* 36-1 showed more regulation related genes (*P* < 0.05), such as enzyme modulators, kinases, membrane traffic proteins, RNA bindings, and transcription factors (Figure 2).

### Weak repeat-induced point mutation (RIP)

The RIP (repeat-induced point mutation) is a genome defense mechanism in fungi that acts against sequence duplication and accumulates G:C to A:T transition mutations (Selker et al., 1987). The RIP index was calculated by using two different indices, namely (CpA+TpG)/(ApC+GpT) and TpA/ApT, which were based on calculations of the dinucleotide frequencies. For the TpA/ApT index, a higher value indicates a stronger RIP, which is the opposite of the (CpA+TpG)/(ApC+GpT) index (Hane and Oliver, 2008). In *P. lilacinum* 36-1, a low average ratio for the TpA/ApT index (0.78) and a high (CpA+TpG)/(ApC+GpT) index (1.51) was observed, indicating weak RIP performance in *P. lilacinum* 36-1. The average ratio of di-nucleotide preference in repeat elements also provided evidence for the weak RIP in *P. lilacinum* 36-1 (Figure S3).

RIP usually occurs during meiosis (Galagan et al., 2003). Two types of compatible MAT genes (*MAT1-1* and *MAT1-2*) are required for sexual development (Kück and Böhm, 2013). The lack of *MAT1-2* which contains the HMG_box domain implied the absence of a sexual phase in *P. lilacinum* 36-1 (Table S4, Figure S4).

RIP impacts on gene duplications, resulting in a decrease in the numbers of gene families (Galagan et al., 2003). A weak RIP could not influence gene expansion in *P. lilacinum* 36-1. We found 166 pairs of duplicated genes with >90% of nucleotide sequence similarities in *P. lilacinum* 36-1 in contrast to only 6 pairs in the RIP-effective species *N. crassa* (Galagan et al., 2003).

### Weak ability to degrade the plant cell walls

**Figure 3:**
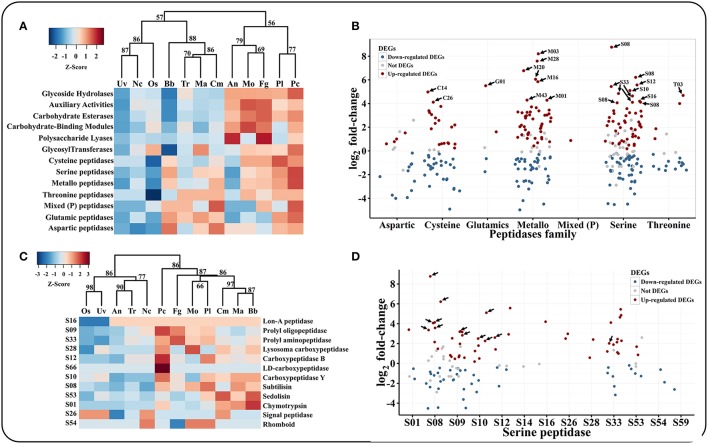
**Comparison and expression patterns of CAZYs and peptidase families in ***P***. ***lilacinum*** 36-1. (A)** Hierarchical clustering of CAZYs and peptidases families in 12 fungi. Uv, *U. virens*; Nc, *N. crassa*; Fg, *F. graminearum*; Os, *O. sinensis*; Bb, *B. bassiana*; Tr, *T. reesei*; Ma, *M. acridum*; Cm, *C. militaris*; An, *A. nidulans*; Mo, *M. oryzae*; Pl, *P. lilacinum*; Pc, *P. chlamydosporia*. Protein families are represented by their family names according to the CAZY and merops database. Approximately unbiased (AU) *P*-values (%) are computed by 1000 bootstrap resamplings by using the R package pvclust. **(B)** Expression profile of different peptidase families. Sub-families with high expression values (log_2_ fold-change > 4) are indicated by arrows. **(C)** Hierarchical clustering of serine peptidase sub-families in 12 fungi. **(D)** Expression profile of serine peptidase sub-families. The arrows indicate signal proteins.

Many plant pathogens require glycoside hydrolases (GHs), carbohydrate esterases (CEs) and polysaccharide lyases (PLs) to degrade the plant cuticle and cell wall. Although *P. lilacinum* 36-1 could not infect plants, it does have the ability to live in the roots of plants as an endophyte (Lopez et al., 2014). A hierarchical clustering analyses showed that *P. lilacinum* 36-1 was most related to *P. chlamydosporia* (Figure 3A), a fungus that acted as an endophyte in monocot and dicot plants.

Cellulolytic enzymes are used in interactions with plants, especially in plant root colonization by endophytic fungi (Strakowska et al., 2014). *P. lilacinum* possessed certain numbers of genes that were involved in cellulose and hemicellulose degradation, in particular β-glucosidase (GH3), cellulases (GH5), endo-β-1,4-glucanase (GH12), β-xylosidase (GH43), α-mannosidase (GH47), hydrolyzing lignocellulose (GH35), cellulose-binding domains (family CBM1), and copper-dependent lytic polysaccharide monooxygenases (family AA9) (Table S5). In comparison with plant pathogenic fungi, however, the absence of cellobiohydrolase I (GH6) and cellobiohydrolase II (GH7) in *P. lilacinum* 36-1 attenuated the capacity for cellulolytic degradation (Table S5). Carbohydrate esterases (CE) cooperate with glycoside hydrolases (GH) in plant polysaccharide degradation to overcome the complexity of the plant cell walls (Biely, 2012). *P. lilacinum* 36-1 contained more CEs in comparison with the entomophagous fungi, but less than those of cereal pathogens *F. graminearum* and *M. oryzae*, such as acetyl xylan esterase (CE1), acetyl xylan esterase (CE2), cutinase (CE5), and arylesterase (CE10) (Table S5), indicating a relatively weaker ability to decompose cell wall. The only exception was that *P. lilacinum* 36-1 had 3 genes, while *M. oryzae* did not have any genes and *F. graminearum* only had one gene of CE7 family (cephalosporin-C deacetylase) (Table S5). Notably, fewer cutinases (CE5) suggested that *P. lilacinum* 36-1 might not depend on direct penetration into the plant roots by degrading the cuticle. Likewise, *P. lilacinum* 36-1 contained much less polysaccharide lyase (PL) than plant pathogens, with a lack of pectin lyase (PL1), pectate lyase (PL3) and rhamnogalacturonan lyase (PL4) (Table S5). Therefore, *P. lilacinum* 36-1 might be unable to degrade pectin effectively during the saprophytic or endogenous phase.

To validate the result discussed above, the capacity of *P. lilacinum* 36-1 to dissociate components of the cell wall (cellulose, pectin, and xylan) was evaluated in comparison with that of *F. graminearum* and *T. virens*. As expected, in comparison with the other two fungi, *P. lilacinum* 36-1 had a weaker ability to break down the three substances as indicated by the smaller halos observed in the medium by using the substances as the only carbon source (Figure S4). Moreover, *P. lilacinum* 36-1 grew better in the medium that was supplied with cellulose than that of mediums containing pectin and xylan, which was consistent with the partial cellulose degradation gene family expansion mentioned above.

### Serine and metallo-peptidases act as dominant factors during nematode-eggs infection

The primary substances of cuticles on nematode eggs are made up of protein-chitin (Wharton, 1980). Thus, nematode eggs pathogens must secrete large amounts of hydrolytic enzymes, such as peptidases or carbohydrate hydrolases, to penetrate the shell and solubilize the host tissues for nutrition (Yang et al., 2007).

*P. lilacinum* 36-1 possessed 333 genes that encoded peptidases, which were more than those of the other sequenced fungi, with 86 putative secreted proteins (Table S6). The most abundant peptidases families were serine peptidases (121 genes) and metallopeptidases (95 genes), which were much more than those of the plant pathogen, with average numbers of 86 and 78, respectively (Table S6). These two peptidase families were considerably up-regulated during infection (Figure 3B).

Among the serine peptidases, the subtilisin family (S08) had the highest relative expansion with 30 genes in *P. lilacinum*, a little more than that in *P. chlamydosporia* (23 genes) and *M. acridum* (27 genes) (Table S6, Figure 3C). Subtilisin (S08) could be induced by nitrogen sources (including nematode cuticle) and play an important role during the infection of nematophagous fungi (Wang et al., 2010; Zou et al., 2010). Consistent with their important functions, 8 subtilisin coding genes were up-regulated and most of them were putative secreted proteins (Figure 3D). The second common serine peptidases were prolyl oligopeptidase (S09) and prolyl aminopeptidase (S33), which could help *P. lilacinum* 36-1 to degrade a proline-rich substrate (Table S6). Both of these two gene family had a different expression level when parasitizing nematode-eggs (Figure 3D). Trypsin (S01) is responsible for degrading the chitin-protein layer of the nematode eggshell and affecting the normal embryogenic development of nematodes (Suarez et al., 2004). However, unlike the entomophagous fungi, *P. lilacinum* 36-1 possessed only 4 genes that coded for trypsin (S01), which was much less than those in *B. bassiana* (14 genes) and *M. acridum* (10 genes) (Table S6), and only one of them was up-regulated at a relatively lower level of expression (Figure 3D).

Despite the serine peptidases, the over-representation of metallopeptidases may also function during infection (Table S6). These families such as aminopeptidase (M03), oligopeptidase (M08), and carboxypeptidase (M20) were particularly up-regulated significantly (log_2_ fold-change > 4) (Figure 3B). In addition, aspartic peptidases which are assist the human pathogen *C. albicans* by degrading cell surface molecules (Schaller et al., 2005) were also an abundant family in *P. lilacinum* 36-1 (Table S6). But this family may not be so activate as metallopeptidases, as indicated by their expression profiles (Figure 3B).

### Chitinases are not the primary factors at the early stage of nematode eggs infections

Based on the CAZYs database (Carbohydrate-Active enZYmes Database), we found that *P. lilacinum* 36-1 had a considerable number of carbohydrate-active enzymes. Glycoside hydrolases (GHs), auxiliary activities (AAs) and carbohydrate esterases (CEs) were well-represented in *P. lilacinum* 36-1 in comparison with entomophagous or saprophytic fungi (Figure 3A).

**Figure 4:**
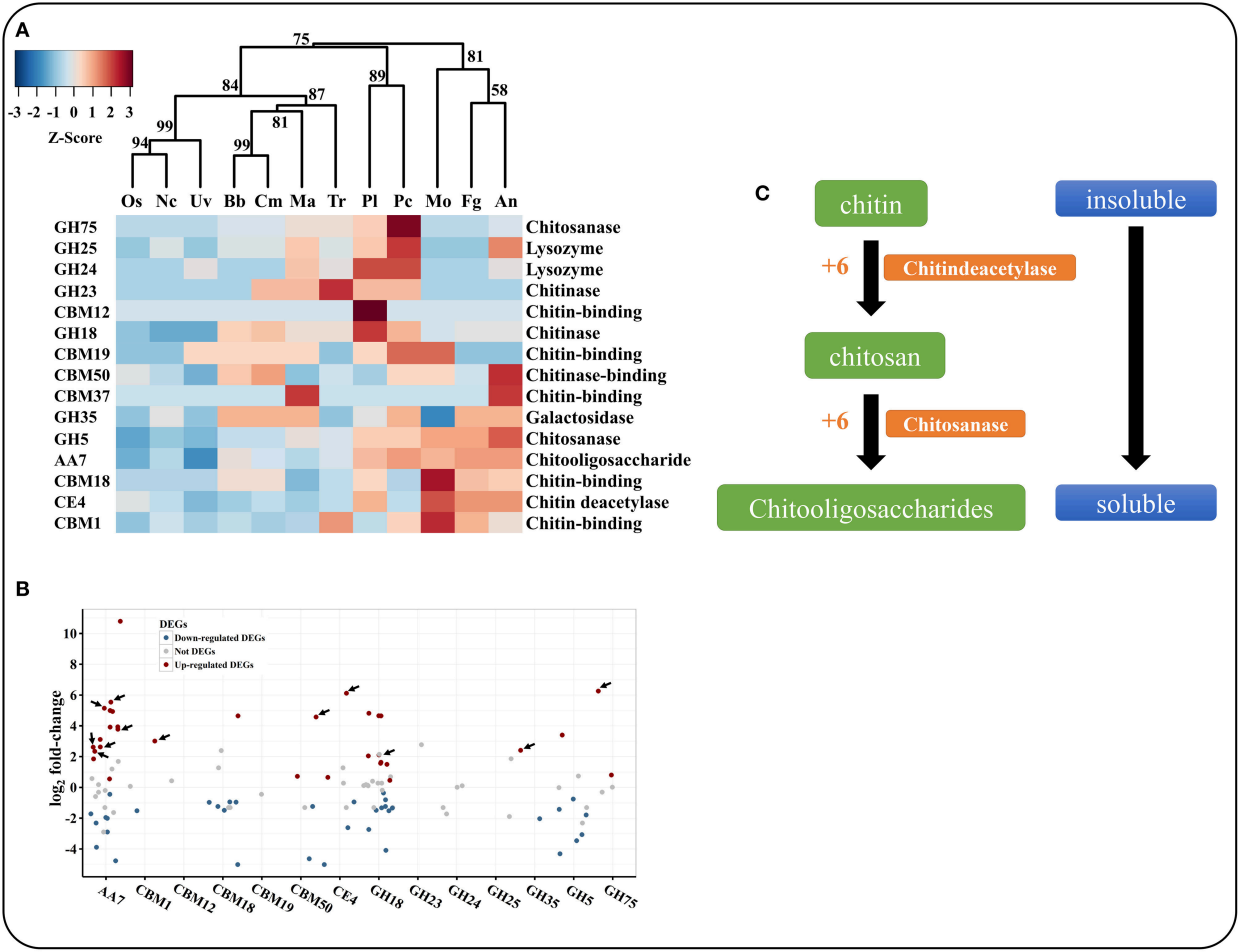
**Comparison and expression pattern of chitin degradation genes in ***P. lilacinum***. (A)** Hierarchical clustering of chitin degradation families in 12 fungi. Uv, *U. virens*; Nc, *N. crassa*; Fg, *F. graminearum*; Os, *O. sinensis*; Bb, *B. bassiana*; Tr, *T. reesei*; Ma, *M. acridum*; Cm, *C. militaris*; An, *A. nidulans*; Mo, *M. oryzae*; Pl, *P. lilacinum*; Pc, *P. chlamydosporia*. Approximately unbiased (AU) *P*-values (%) are computed by 1000 bootstrap resamplings by using the R package pvclust. **(B)** Expression profile of different chitin degradation genes during early stage of *P. lilacinum* infection nematode-eggs. The arrows indicate signal proteins. **(C)** The putative pathway of *P. lilacinum* degradation chitin during infection nematode-eggs. The numbers indicates expression values by transcriptom analysis.

Chitin is the primary component of nematode eggs. *P. lilacinum* 36-1 needs to secrete many chitin-degradation genes to digest nematode egg-shells, such as GHs, CBMs, AAs, and CEs. These types of enzymes exhibited clearly different distributions in various fungi (Table S5, Figure 4A). Hierarchical clustering showed an obvious expansion of chitin degradation genes in *P. lilacinum* 36-1 such as chitinases (GH18), isozymes (GH24, GH25), chitosanase (GH75), chitooligosaccharide oxidase (AA7) and chitin deacetylase (CE4) in *P. lilacinum* 36-1, suggesting a strong ability to degrade chitin (Figure 4A). During the early stage of nematode eggs infections, these genes were up-regulated at different levels, indicating their importance in nematode eggs-shell degradation (Figure 4B). Moreover, in considering the crucial functions of secretory proteins, we predicted the sub-cellular localizations of these up-regulated genes. Surprisingly, only one chitinase gene belonged to extracellular protein but it had a relatively lower expression value (Figure 4B). By contrast, the secretory proteins CE4, GH75, AA7 and CBM50 (chitin-binding) exhibited a high expression value (Figure 4B). These results suggested that the degradation of chitin in nematode egg-shells might not primarily depend on the chitinase hydrolysis pathway, but on successive processes of chitin deacetylase, chitosanase, and chitooligosaccharide oxidase assisting by chitin binding protein, thus making the egg-shell soluble enough for *P. lilacinum* 36-1 infection at the early stage (Figure 4C). In particular, the chitin deacetylase in *P. lilacinum* 36-1 was homologous with an appressorium differentiation related gene (UniPortKB: D1MYV6) in *M. oryzae*, indicating that the up-regulated chitin degradation genes might be involved in appressorium formation at the early infection stage.

### Abundance of transporters assist broad environmental adaptation of *P. lilacinum* 36-1

Membrane transporters are involved in defending against toxic metabolites and in transporting nutrients (Costa et al., 2014). Based on a BLAST search against the TCDB database, we identified 614 transporters in the *P. lilacinum* 36-1 genome. Interestingly, the transporter content was similar to that of *F. graminearum*, but much higher than that of entomophagous and saprophytic fungi (Table S7), including the major facilitator superfamily (MFS), amino acid-polyamine-organocation family (APC), drug/metabolite transporter superfamily (DMT), and ATP-binding cassette superfamily (ABC). Considering the closer genetic relationship and the huge differences in inhabited environments between *P. lilacinum* and *O. sinensis*, we concluded that the high transporter genes content were the key factors of *P. lilacinum* 36-1 to adapt diverse environments.

More than 160 transporters were significantly up-regulated (log_2_ fold-change > 2) when parasitizing nematode-eggs (Table S8). In particular, ~101 genes encoding MFS were up-regulated during nematode eggs infection (Table S8), including the sugar porter (SP) family (2.A.1.1), the anion: cation symporter family (2.A.1.14), drug: H^+^ Antiporter-1 family (2.A.1.2), and drug: H^+^ Antiporter-2 family (2.A.1.3), suggesting the strong nutrition-related metabolism of *P. lilacinum* 36-1 at this stage. Amino acid transporters react at the initial parasitic stage of nematophagous fungi (Rosso et al., 2011). Consistent with their important roles, 16 APC transporters (2.A.3) were significantly up-regulated during *P. lilacinum* 36-1 infection, especially for arginine permease (2.A.3.10.4) with its eight-fold higher expression level (Table S8). ABC transporters facilitate plant parasitic fungal infection by protecting them from antifungal compounds (Gardiner et al., 2013). Twelve ABC transporter genes were up-regulated, half of which belonged to the pleiotropic drug resistance family (PDR). Moreover, the Pi uptake porter (2.A.1.9.2) and Pi-repressible Pi:Na+ symporter (2.A.20.2.1), in response to the availability of phosphorus under phosphorus-limiting conditions (Versaw, 1995), had remarkable expression (11 and 9-fold, respectively), indicating that *P. lilacinum* 36-1 might suffer phosphate-restrictive conditions when infecting nematode eggs.

### Signal transduction genes involved in nematode eggs infection

G protein–coupled receptors (GPCRs), which are also known as seven-transmembrane domain receptors, are only found in eukaryotes. They constitute a large protein family of receptors that sense molecules outside the cell and activate signal transduction pathways, and ultimately cellular responses (Trzaskowski et al., 2012). In fungi, they are required for pheromone/nutrient sensing and host recognition (Gao et al., 2011). *P. lilacinum* 36-1 had 70 genes coding for GPCRs in comparison with the insect pathogen *M. acridum* with 57 genes (Table S9). Two genes encoding STM1, a recognition molecule for nitrogen starvation signals (Chung et al., 2001), were predicted in *P. lilacinum* 36-1. One of them was up-regulated, which suggested that the fungi may face nitrogen-limited environment during infection, consistent with the insect parasitic process of *Metarhizium spp* (Gao et al., 2011). In addition, the up-regulation of genes encoding the cAMP receptor indicated that *P. lilacinum* 36-1 might go through the energy metabolism process. Notably, *PTH11* is a pathogenicity gene that was predicted to encode a transmembrane protein and was related to host surface recognition and appressorium formation in *Magnaporche oryzae* (DeZwaan, 1999). *P. lilacinum* 36-1 had 53 *PTH11*-like genes, as many as that of *M. anisopliae* (Table S9) and 24 of them were up-regulated, indicating their key role during nematode-egg infection (Figure S6).

In fungi, G protein alpha subunits play essential roles during sexual and pathogenic development through the transduction of extracellular signals to affect morphogenetic processes (Tan et al., 2009). Four classes of G protein alpha subunits play distinct roles (Kamato et al., 2015). *P. lilacinum* 36-1 contained four G-alpha genes. The up-regulated genes *PCL_06420* and *PCL_11517* showed the best hits (> 35% protein sequence similarity) with *MAA_05603* (GPA2) and *MAC_04984* (GPA1), both of which were also up-regulated during infection in *Metarhizium* spp. (Gao et al., 2011), suggesting that a similar signal transduction pattern was involved in the pathogenicity between the egg-parasitic and entomaphagous fungi.

Following extracellular recognition, the downstream kinase pathway was initiated. *P. lilacinum* 36-1 possessed 139 protein kinases, 18 of which were up-regulated (log_2_ fold change > 2) during infection (Figure S5). Moreover, the first six highly expressed protein kinase coding genes during this stage were serine/threonine-protein kinases (Figure S5), indicating their potential roles. Like *Metarhizium spp*., the increased transcript abundance of the G-protein alpha subunit, phosphatidylinositol-specific phospholipase C, calcium/calmodulin-dependent protein kinase, and protein kinase C indicated the mitogen-activated protein kinase (MAPK) pathway was strongly activated in *P. lilacinum* 36-1 in the early *P. lilacinum*-eggs interactions (Table S10).

### *P. lilacinum* 36-1 is rich in antibiosis effector-like proteins

Secreted proteins, especially for effectors, play pivotal roles in pathogenicity (Dodds et al., 2009). The *P. lilacinum* 36-1 genome encoded 879 signal peptides (without transmembrane domains), of which 279 were potential effector-like proteins (length: < 300 bp; cysteine: ≥4) (Table S11). Based on the InterProScan analysis, we compared the numbers of effector-like proteins with the particular function. Despite the hydrolases and signal molecule genes mentioned above, *P. lilacinum* 36-1 encoded more toxin-like polypeptides than other organisms (Table S11, Figure S7), such as lysozyme, for degrading the bacterial cell walls (Samaranayake et al., 2009) and Kp4, for inhibit cell growth and division by blocking calcium import into mammalian cells (Park et al., 1994) as well as ribonuclease, for inhibiting protein biosynthesis and then resulting in cell death by apoptosis (Lacadena et al., 2007). Both the Kp4 and ribonuclease encoding gene were up-regulated at the early nematode eggs infection (Table S12). In addition, the presence of two aerolysin/hemolysin toxins, bacterial toxins that disrupts human erythrocytes declined (Buckley et al., 1995) (Table S11), which indicated the potential virulence of *P. lilacinum* 36-1 to humans. Notably, there were eight extracellular membrane proteins with CFEM domains and three of them were up-regulated indicating their importance in the pathogenicity to nematode-eggs (Tables S11, S12).

### High-level expression of genes related to appressorium formation during nematode-eggs infection

Like *Metarhizium spp., P. lilacinum* 36-1 could form appressorium when infecting nematode eggs (Khan et al., 1999). Based on a BLASTP search against appressorium-related genes as retrieved from the UniPort database at an evalue of 1e-05, we found 357 analogous genes in *P. lilacinum* 36-1, which were more than those in entomophagous fungi, but less than in plant pathogens (Table S13). Curiously, there were little differences among nematophagous fungi, entomophagous fungi, phytopathogens, or saprophytes. All these fungi have most of the appressorium-related genes, except for the Magas2 protein-coding gene (UniPortKB: G3G3V4), which was exclusively present in nematophagous/entomophagous fungi (Table S13). This gene was found to be predominantly expressed in the appressorium-formation stage in *M. acridum*, but its function was still not well-documented (Zhu et al., unpublished).

During the early infection stage of *P. lilacinum* 36-1, 68 appressorium formation-related genes were up-regulated (Table S14), suggesting a strong metabolite involved in appressorium-formation. Notably, There were 27 genes having a comparatively high expression level (log_2_ fold-change >4) (Table S14), which might be used as potential candidate genes in future research.

### *P. lilacinum* 36-1 showed obvious metabolic processes turnover during nematode eggs infections

**Figure 5:**
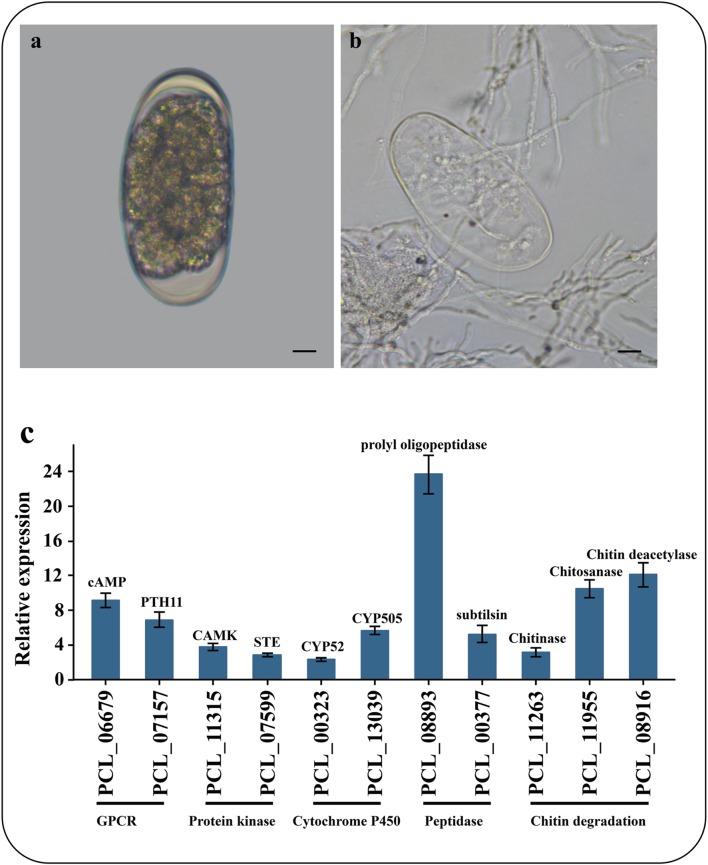
**Microscopic observation and qPCR of genes involved in infection. (A)** Un-infection nematode-eggs. **(B)** Infected eggs by *P. lilacinum* 36-1. Bar indicate 10 μm. **(C)** qPCR of genes involved in infection. Error bar represent the stand error of averages from four technology replicates.

To identify the genes associated with the infection processes, we used RNA-Seq to compare the gene expression profiles and to identify the difference between non-infection mycelia and mycelia supplied with nematode-eggs as the only nourishment for 24 h. The infections were confirmed under a light microscope (Figures 5A,B). As a result, more than 93% of the reads were mapped to the genome by TopHat2. Relative to the results during growth in water, 2603 genes were significantly up-regulated while 2428 genes were remarkably down-regulated during the parasitism process (GFOLD < 0.01, |log_2_ fold-change| > 2). And some critical up-regulated genes were confirmed by qPCR (Figure 5C).

**Figure 6:**
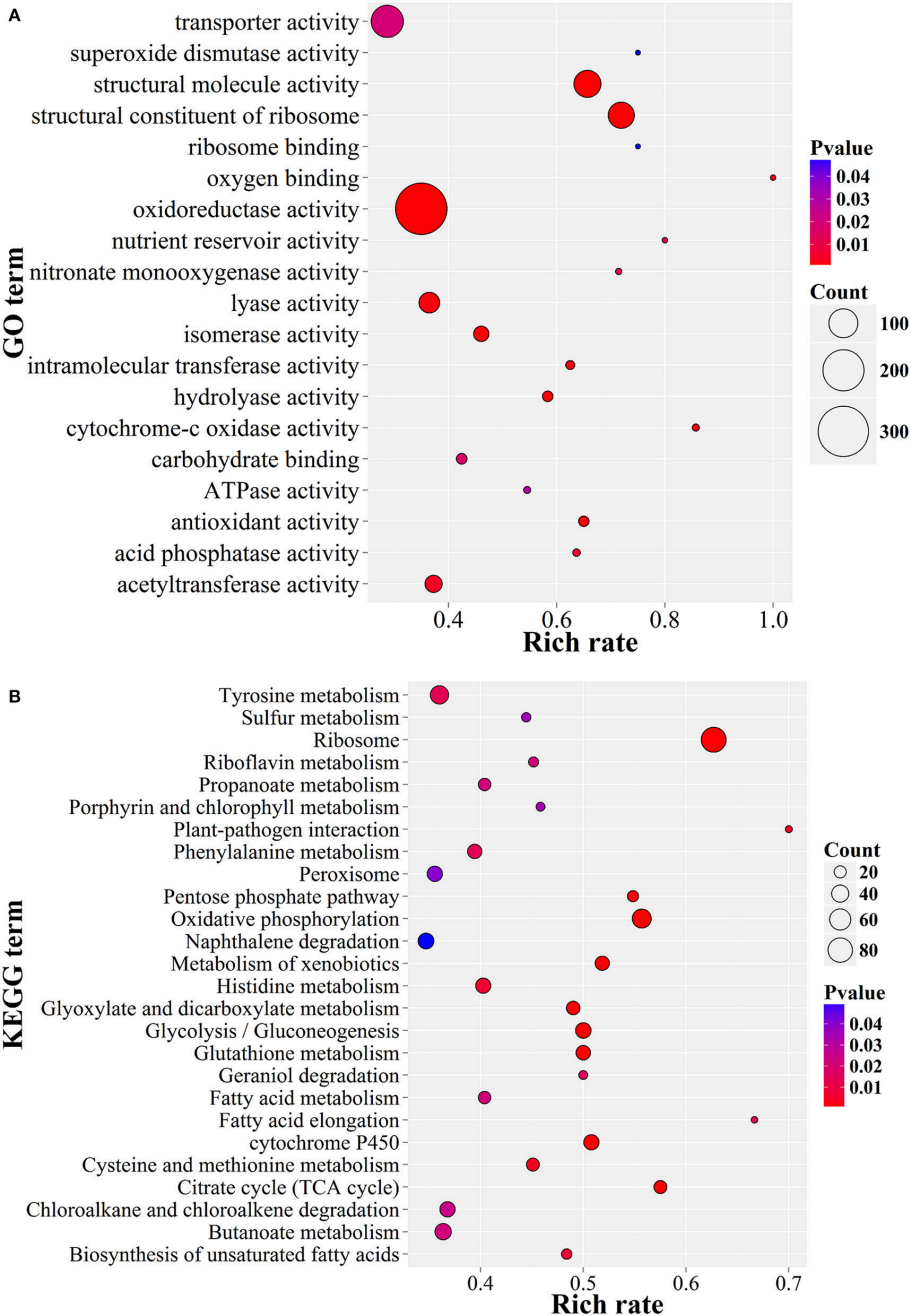
**GO and KEGG enrichment of ***P. lilacinum*** 36-1 infection nematode-eggs. (A)** Gene ontology enrichment of up-regulated genes. **(B)** KEGG enrichment of up-regulated genes. Count represents the numbers of genes enriched in a particular GO or KEGG term. The bigger the value of rich rate and the smaller the value of *P* value indicate the degree of enrichment is more significant.

The GO enrichment results showed that the up-expressed genes related to oxidoreductase activity, structural constitutes of ribosome and transferase activity, as well as transmembrane transporter activity were enriched (*P* < 0.05; Figure 6A). Consistently, these genes were also significantly enriched in serial energy metabolism-related processes, especially for ribosome biogenesis, oxidative phosphorylation and amino acid metabolism (*P* < 0.01; Figure 6B). Moreover, the up-regulated genes were also enriched in glutathione metabolism, glycolysis and the pentose phosphate pathway. These results suggested a high turnover of metabolic processes during appressorium formation, which was consistent with the observation in *M. oryzae* (Oh et al., 2008). A similar situation had also been observed in nematophagous/entomophagous fungi, mycoparasites, or human pathogens (Rosso et al., 2011). In addition, the enrichment of the plant-pathogen interaction pathway (Figure 6B) indicated that *P. lilacinum* 36-1 might implement a similar strategy as phytopathogens to establish successful infections.

As in *Metarhizium spp* (Gao et al., 2011), the genes in response to stress were also highly up-expressed, such as superoxide dismutase, HSP20 and HSP30, cytochrome c oxidase, glutathione S-transferase, and superoxide dismutase (Figure 6, Table S15). In addition, Zn (2)-C6 fungal-type DNA-binding and C2H2 transcription factors were also involved in the infection process of *P. lilacinum* 36-1 (Table S10). Furthermore, gene families such as catalase and superoxide dismutase suppressing the host defense responses and crotonases involved in fatty acid metabolism (Piekarska et al., 2006) were well-explored as virulence factors. These genes were also up-expressed during nematode-eggs infection on the basis of the RNA-Seq analyses (Table S15), all of which could be putative pathogenicity determinants in *P. lilacinum* 36-1.

## Discussion

In this study, we sequenced the genome of a nematode-egg parasitic fungus *P. lilacinum* 36-1 and further investigated the early responses of the fungus to *Meloidogyne incognita* eggs. The resultant genome was more compact and dynamic than that of the *P. lilacinum* strain sequenced before, which was isolated from tannery sludge in India (Prasad et al., 2015).

A phylogenetic analyses showed that *P. lilacinum* 36-1 was closer to *O. sinensis*, a strictly obligate parasite (Hu et al., 2013). Moreover, these two fungi shared fewer orthologous genes than those between *P. lilacinum* 36-1 and *P. chlamydosporia, M. acridum, F. graminearum* (Figure 1C). In comparison with *O. sinensis, P. lilacinum* 36-1 contained much more carbohydrate-active enzymes, peptidases, transporters, and signal transduction genes (Figure 2). In considering the huge difference in living environments between *P. lilacinum* 36-1 and *O. sinensis*, we speculate that these two fungi may undergo an obvious direction in their evolutionary processes, where *P. lilacinum* tends to more broad in its habitat scope while *O. sinensis* is concentrated. The expansion of gene families in *P. lilacinum* 36-1 may be related to the lower level of repeat-induced point mutations (RIP). *P. lilacinum* had orthologs (*PCL_12430*) with the *N. crassa* RIP defective gene RID (*E* < 10^−10^), the only gene known for RIP (Freitag et al., 2002). The retention of this gene in *P. lilacinum* 36-1 indicates that RIP might play roles at some developmental stages during its evolution even though the genome showed no apparent transitions from C:G to T:A. The RIP has a strong correlation with meiosis during sexual reproduction (Hane and Oliver, 2008). However, the sexual stage of *P. lilacinum* 36-1 is still unknown. In fact, it is reported that the predominant mode of reproduction in *P. lilacinum* 36-1 is clonal reproduction in nature (Li et al., 2013). Even so, *P. lilacinum* 36-1 possessed apparent functional genes whose orthologs in *N. crassa* and *A.nidulans* are known to be required for meiosis and sexual development (Table S4), suggesting that *P. lilacinum* 36-1 may have potential for homothallism or heterothallism under appropriate conditions. The expansion of gene families, lower RIP and loss of sexuality are associated with the transformation from specialization to generalization in *Metarhizium* spp. (Hu et al., 2014). This mechanism may apply to *P. lilacinum* 36-1 as indicated by the characteristics of its genome and increased phenotypic flexibility (nematophagous, endogenous, and saprophytic) (Table 2), demonstrating and enlarging the applicability of the theory.

**Table 2:** **Comparison of selected protein families between ***P. lilacinum*** 36-1 and other fungi**.

**Features**	**36-1**	**Pc**	**Os**	**Ma**	**Fg**	**Mo**	**An**	**Nc**
Trophic type	N/S/End	N/S/End	Ent	Ent	P/S	P/S	S	S
Trypsin	4(1)	7(7)	1(0)	10(10)	3(2)	2(3)	2(2)	1(0)
Subtilisin	30(16)	23(19)	11(9)	27(20)	17(14)	24(15)	3(1)	5(3)
Metallopeptidase	95(16)	108(20)	52(12)	76(10)	89(6)	88(9)	77(10)	60(4)
Glycoside hydrolase	256(77)	303(103)	90(32)	171(56)	260(98)	262(127)	264(112)	188(51)
Chitinase	24(8)	18(8)	12(1)	15(6)	17(6)	14(6)	17(2)	9(0)
Fungal chitosanase	4(3)	11(10)	1(1)	3(2)	1(0)	1(1)	2(1)	1(0)
Chitin-binding	11(4)	6(3)	5(1)	1(0)	16(10)	27(20)	15(5)	3(0)
Pectin lyase	8(4)	11(10)	2(1)	4(4)	26(22)	9(5)	26(21)	12(4)
Cutinase	5(4)	6(6)	2(2)	2(2)	12(10)	18(15)	4(4)	3(2)
Cellulose-binding	3(3)	9(8)	0(0)	2(1)	12(7)	22(18)	7(6)	18(14)
Protein kinase	139	139	174	192	149	135	139	119
Extracellular membrane protein CFEM	32(12)	28(9)	7(2)	11(4)	20(5)	20(9)	6(4)	9(6)
Cytochrome P450	93	123	56	101	117	141	126	47
Transcription factor	235	254	70	162	240	135	235	109

*N: nematophagous; S: saprophytic; End: endophytic; Ent: entomophagous; P: phytopathogen; Numers in bracket represent signal proteins. Pc, Pochonia chlamydosporia; Os, Ophiocordyceps sinensis; Ma, Metarhizium acridum; Fg, Fusarium graminearum; Mo, Magnaporthe oryzae; An, Aspergillus nidulans; Nc, Neurospora crassa*.

The parasitic processes of these fungi include the following successive steps: attraction or recognition, adhesion, penetration, and digestion (Qiaozhen et al., 2009). However, the mechanism through which fungi recognize nematodes and initiate morphological transition remains unknown. Unlike nematode-trapping fungi that sense volatile organic compounds (VOCs) produced by nematodes (Dong and Zhang, 2006), egg-parasitic fungi recognizing nematode-eggs may primarily depend on physical contact due to obstruction of VOCs by egg-shells, requiring signal transduction sensors to perceive environmental cues and thus transfer the signals into intracellular spaces. Extracellular membrane proteins containing an eight-cysteine domain known as CFEM were identified as signal molecules (cell-surface receptors or adhesion molecules) in plant pathogenic fungi (Kulkarni et al., 2005). *P. lilacinum* 36-1 contained much more CFEM-containing proteins (32) than *M. acridum* (11) and *M. oryzae* (20) (Table 2), and one gene had a high expression value of 7.6-fold (Table S15), suggesting the importance of CFEM protein during the recognition of nematode-eggs. Consistent with *Metarhizium spp*. (Gao et al., 2011), the differential expression of GPCR (*PTH11* and *STM1*) genes may also take part during the recognition process. All these genes could be candidates for further analyses to investigate their function as pathogenicity regulators.

After successful recognition, the nematophagous fungi attach to their hosts via adhesive proteins. Lectins were once considered responsible for adhesive traps in nematode-trapping fungi (Li et al., 2015). Although recent research showed that lectins were not necessary for pathogenicity of nematode-trapping fungi *A. oligospora* against nematodes (Balogh et al., 2003), the up-regulation of lectin-coding genes when *P. lilacinum* 36-1 infecting nematode-eggs showed that this protein may be involved in the pathogenicity of egg-parasitic fungi (Table S15). Along with lectins, *mad1* is the only gene known to regulate the attachment of the entomopathogenic fungus *M. anisopliae* to insects (Wang and St. Leger, 2007). In *P. lilacinum* 36-1, PCL_01111 is homologous to *mad1* genes (similarity: 45%; *E*-value: 2e-149). The up-regulation of *PCL_01111* (log_2_ fold-change: 4.8-fold) suggests that it may mediate the adhesive processes during the early stage of egg-parasitic fungi infection of nematode-eggs. Unexpectedly, the homologous gene *PCL_12454* in *P. lilacinum* 36-1 (protein sequence similarity: 45%; *E*-value: 2e-53) with *mad2*, which mediates attachment to plants but not insects in *M. anisopliae*, was also up-regulated (log_2_ fold-change: 3-fold). These results reflect the intercommunity and specialization of the adhesion mechanism between egg-parasitic and entomopathogenic fungi.

Egg-parasitic fungi depend on appressoriums, penetration pegs, or lateral mycelial branches to infect nematode eggshells (Li et al., 2015). In comparing *P. lilacinum* 36-1 with other fungi, we found that most of the appressorium-forming related genes were ubiquitous, even in fungi lacking ability to form appressoriums (Table S13). The conservation of these genes suggests that they are superior regulation mechanisms for mediating appressorium-forming, even though signal transduction genes are included (Table S13). This hypothesis was further supported by the common activation of MAPK and *PTH11-like* GPCR in *Magnaporthe grisea* (Gupta and Chattoo, 2007), entomopathogenic (Gao et al., 2011) and egg-parasitic fungus *P. lilacinum* 36-1.

Because of the chitin and protein components of nematode eggs-shells, extracellular hydrolytic enzymes, such as the chitinases and peptidases in egg-parasitic fungi are required for disintegrating eggshell layers (Yang et al., 2007). Serine peptidases are believed to play important roles in nematode eggs infection (Wang et al., 2010). In comparison with other fungi, *P. lilacinum* 36-1 contained large numbers of serine peptidases (Figure 3C). Consistent with their vital function, many of them were up-regulated, including S08, S10, S12, S16, and S33 subfamilies (Figure 3D). Moreover, in accordance with a previous report about egg-parasitic fungi *Trichoderma harzianum* (Szabo et al., 2013), cooperation of different peptidase families was also observed in *P. lilacinum* 36-1, as indicated by the up-regulation of aspartic peptidase, metalloendopeptidases and the most prominently induced serine peptidases (Figure 3B). Likewise, chitin degradation was also found to have co-expression with peptidases (Yang et al., 2007). To date, most of researches about chitin degradation genes in egg-parasitic fungi focus on the latter infection stages (Dong et al., 2007; Cletus et al., 2013). Particularly, the critical virulence factors chitinase GH18s (Tikhonov et al., 2002) were well-represented (24) and eight of them were predicted to be putatively secreted in *P. lilacinum* 36-1. However, although many GH18s were up-regulated during nematode-eggs infection, they may be not dominant virulent factors as indicate by only one signal protein which has a relatively lower-level of expression at the same time (Figure 4B). In fact, the high-level expression of chitin deacetylase, chitosanase, and chitooligosaccharide oxidase suggest that these enzymes may play key roles in degrading the chitin of nematode egg-shells during infection assisting by chitin binding related genes (Figure 4C). Some of the genes mentioned above were verified by qPCR (Figure 6B). Notably, chitin deacetylase (CE4) was also found to be involved in appressorium forming, which was consistent with the results mentioned above.

Interestingly, *P. lilacinum* 36-1 possessed certain numbers of genes related to the degradation of cellulose and xylan, but fewer genes encoding polysaccharide lyases (Table S5). These substances are the primary components of the plant cell walls. This finding suggests that the fungi are able to employ living plant tissues and potential ability to colonize the plant root surface, which is consistent with the frequency isolated of *P. lilacinum* from the rhizosphere (Lopez et al., 2014). Considering the sedentary plant nematodes and some parasitic migration nematodes infecting the host through roots (Jones et al., 2013), we speculate that there might be a co-evolutionary relationship among *P. lilacinum*, host plants and nematodes.

## Conclusions

In this study, we identified the genome structure, gene contents and transcriptional regulations to nematode eggs of *P. lilacinum* 36-1. Comparative genomic showed an obvious abundance in peptidases, carbohydrate enzymes, transporters and signal transduction genes with particular functions related to pathogenicity. Furthermore, the transcriptomic analyses indicated that the infection patterns of nematode-eggs parasitic fungi were similar to those of *Metarhizium* spp. parasitizing insects. The genomic and transcriptomic sequences will facilitate to identifying candidate genes involved in the interactions between nematophagous fungi and nematode-eggs and further to promote practical applications in bio-control.

### Nucleotide sequences accession numbers

The whole project sequences were deposited in the DDBJ/EMBL/GenBank repository under BioProject PRJNA281297. The complete genome sequence is available under accession number LCWV00000000. The clean reads have been deposited at NCBI's Sequence Read Archive under accession numbers SRR2536611 and SRR2536612. The Illumina mate-pair reads for RNA-Seq were deposited at the NCBI Sequence Read Archive under accession number SRR2166047.

## Author contributions

YX and GW initiated and designed the research; SL annotated genes, performed protein family analysis, phylogenetic, and genome assembly; JX and CM preformed transcriptome analysis; JX incubated the fungi and nematodes and performed qPCR; JX and SL wrote the paper; DP, GW, and XX reviewed the paper. All the authors read and approved the final manuscript.

## Funding

This project was supported by the Special Fund for Agro-scientific Research in the Public Interest 201503114, the Major State Basic Research Development Program (973) 2013CB127504, and Fundamental Research Funds for the Central Universities 2662016PY044.

### Conflict of interest statement

The authors declare that the research was conducted in the absence of any commercial or financial relationships that could be construed as a potential conflict of interest.

## Abbreviations

RIP: repeat-induced point mutation
ABC: ATP-binding cassette
GH: glycoside hydrolase
GPCR: G-protein coupled receptor
MAPK: mitogen-activated protein kinase
MFS: major facilitator superfamily
GO: gene ontology
KEGG: Kyoto encyclopedia of genes and genomes
qPCR: quantitative real time polymerase chain reaction.
