# Supplementary material for: Genome and Transcriptome Sequences Reveal the Specific Parasitism of the Nematophagous Purpureocillium lilacinum 36-1
Source: Front Microbiol. 2016 Jul 19;7:1084. doi: 10.3389/fmicb.2016.01084 (PMC4949223; doi:10.3389/fmicb.2016.01084)
Supplement: Supplementary file 16 [file Image1.pdf]

Supplementary figure 1

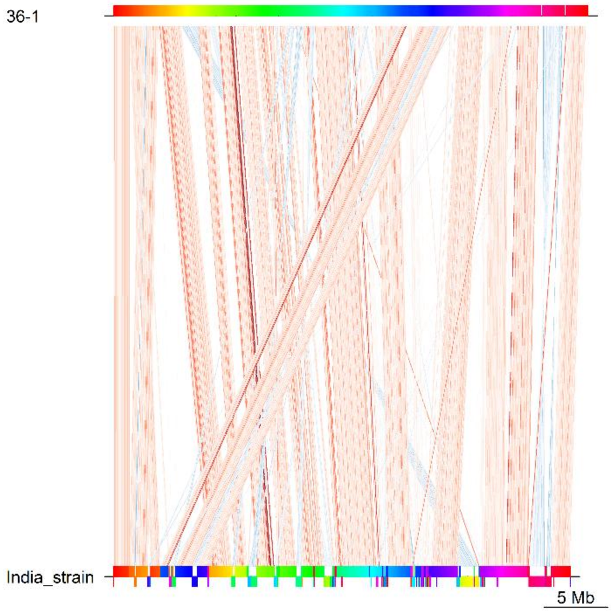

Fig. S1: The whole genome alignment between *P. lilacinum* 36-1 and the India_strain. The line indicats the locally collinear blocks (LCB) (>100 bp). Red lines indicate forward alignments. Blue lines indicate reverse alignments.
