# Supplementary material for: Genome and Transcriptome Sequences Reveal the Specific Parasitism of the Nematophagous Purpureocillium lilacinum 36-1
Source: Front Microbiol. 2016 Jul 19;7:1084. doi: 10.3389/fmicb.2016.01084 (PMC4949223; doi:10.3389/fmicb.2016.01084)
Supplement: Supplementary file 17 [file Image2.PDF]

Supplementary figure 2

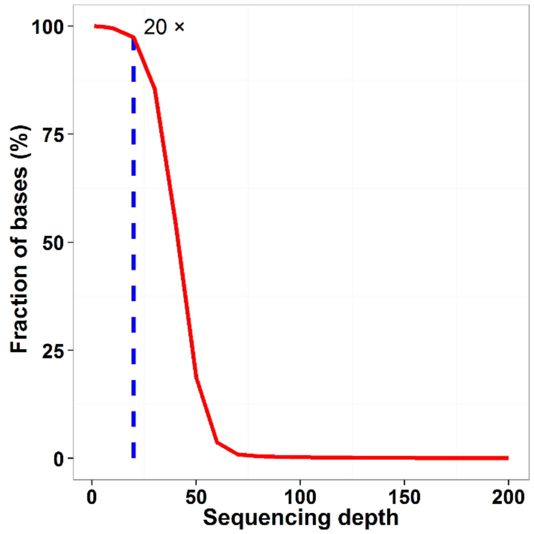

Fig. S2: The alignment information of reads mapping to the genome assembly of *P. lilacinum* 36-1. X-axis denotes the sequencing depth. Y-axis denotes the fraction of bases.
