# Supplementary material for: Genome and Transcriptome Sequences Reveal the Specific Parasitism of the Nematophagous Purpureocillium lilacinum 36-1
Source: Front Microbiol. 2016 Jul 19;7:1084. doi: 10.3389/fmicb.2016.01084 (PMC4949223; doi:10.3389/fmicb.2016.01084)
Supplement: Supplementary file 19 [file Image4.pdf]

Supplementary figure 4

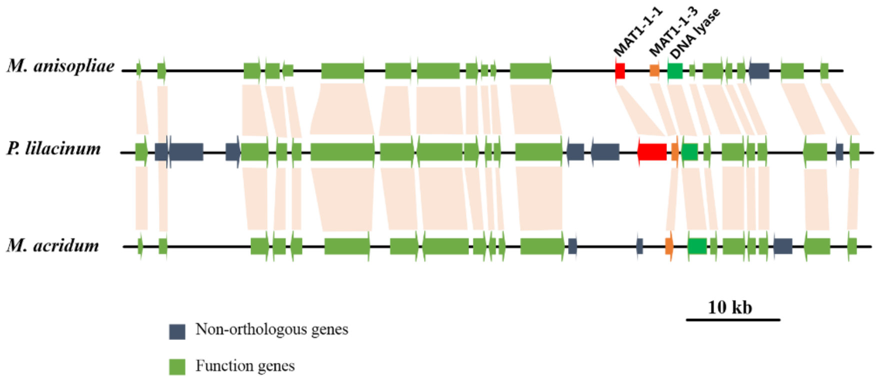

Fig. S4: Collinearity analysis of sexual differentiation gene cluster in *P. lilacinum* 36-1 and *Metarhizium spp.*.
