# Supplementary material for: Genome and Transcriptome Sequences Reveal the Specific Parasitism of the Nematophagous Purpureocillium lilacinum 36-1
Source: Front Microbiol. 2016 Jul 19;7:1084. doi: 10.3389/fmicb.2016.01084 (PMC4949223; doi:10.3389/fmicb.2016.01084)
Supplement: Supplementary file 20 [file Image5.PDF]

Supplementary figure 5

Cellulose **Xylan** **Pectin**

36-1

T. reesei

F. graminearum

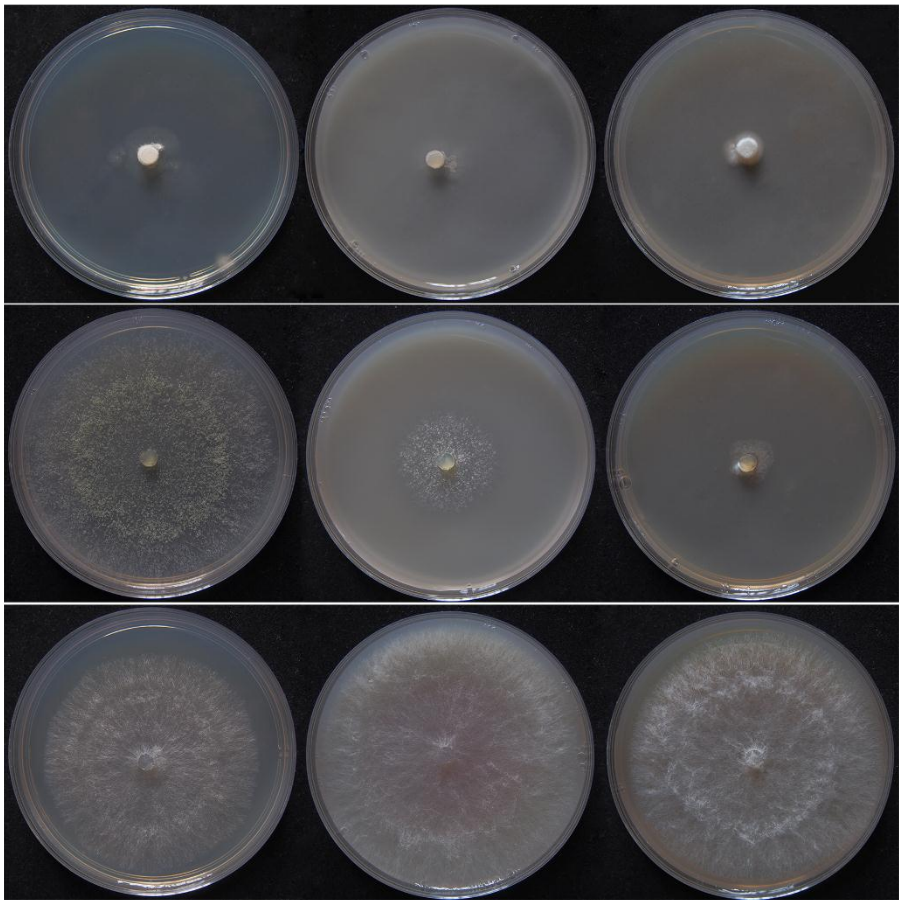

Fig. S5: Comparison of growth test on different component of cell well between P. lilacinum 36-1 and other fungi. The basic MM medium (1g L^{-1} NH_4NO_3, 0.5g L^{-1} KH_2PO_3, 1.5g L^{-1} K_2HPO_3, 1.0 g L^{-1} NaCl and $MgSO_4.7H_2O$) was supplemented with 5g L^{-1} cellose, xylan or pectin. The fungi were incubated for three days. The experiments were repeated three times.
