# Supplementary material for: Genome and Transcriptome Sequences Reveal the Specific Parasitism of the Nematophagous Purpureocillium lilacinum 36-1
Source: Front Microbiol. 2016 Jul 19;7:1084. doi: 10.3389/fmicb.2016.01084 (PMC4949223; doi:10.3389/fmicb.2016.01084)
Supplement: Supplementary file 21 [file Image6.PDF]

Supplementary figure 6

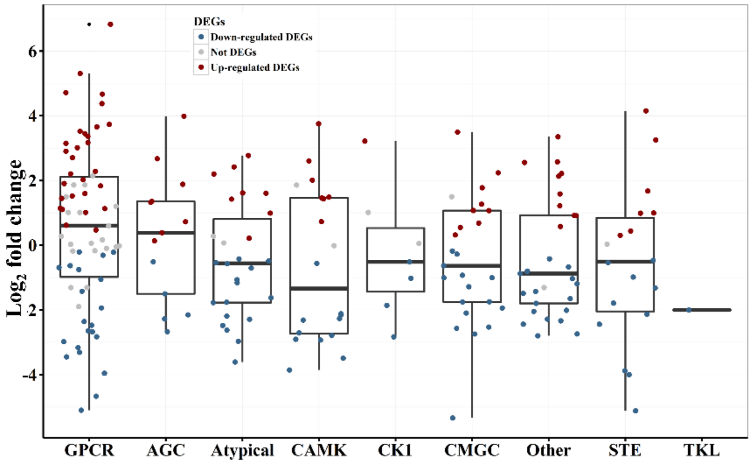

Fig. S6: Expression profiles in signal transduction genes.

GPCR: Guanosine-binding Protein Coupled Receptor; AGC: protein kinase A, G and C group; Atypical: Hisk (histidine kinase), BRD (bromodomain containing kinase), PGHK (pyruvate dehydrogenase kinase); CAMK: Calcium/Calmodulin regulated kinases; CK1: Casein Kinase 1 group; CMGC: CDK (cyclin dependent kinase), MAPK (mitogen-activated protein kinase), GSK3 (glycogen synthase 3 kinase), CLK (CDC-like kinase); Other: other kinase; STE: MAP kinase cascade kinases; TK: Tyrosine kinase; TKL: Tyrosine kinase-like group.
