# Supplementary material for: Genome and Transcriptome Sequences Reveal the Specific Parasitism of the Nematophagous Purpureocillium lilacinum 36-1
Source: Front Microbiol. 2016 Jul 19;7:1084. doi: 10.3389/fmicb.2016.01084 (PMC4949223; doi:10.3389/fmicb.2016.01084)
Supplement: Supplementary file 22 [file Image7.PDF]

Supplementary figure 7

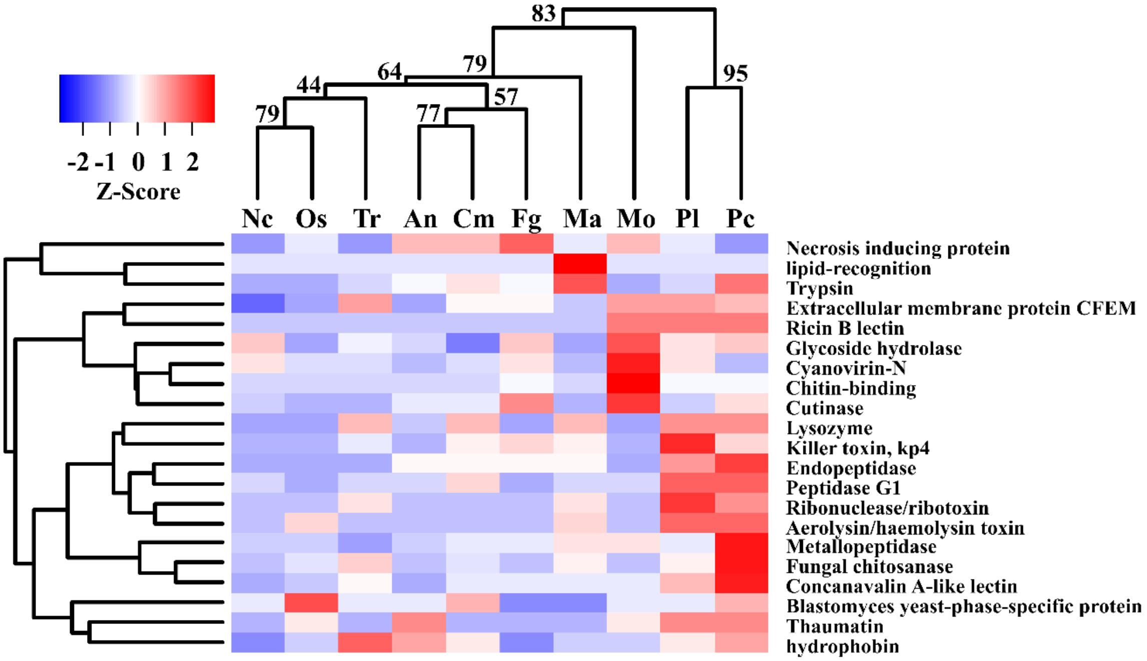

Fig. S7: Effector-like genes in *P. lilacinum* and other fungi.

Uv, *U. virens*; Nc, *N. crassa*; Fg, *F. graminearum*; Os, *O. sinensis*; Bb, *B. bassiana*; Tr, *T. reesei*; Ma, *M. acridum*; Cm, *C. militaris*; An, *A. nidulans*; Mo, *M. oryzae*; Pl, *P. lilacinum*; Pc, *P. chlamydosporia*. Approximately unbiased (AU) P-values (%) are computed by 1000 bootstrap resamplings by using the R package pvclust.
